# Spatial match analysis of multiple factors in the geopolitical environment of the Arctic Passage

**DOI:** 10.1371/journal.pone.0270262

**Published:** 2022-07-01

**Authors:** Chunjuan Wang, Dahai Liu, Jinpeng Wang, Yimeng Zhao, Haiyan Shan

**Affiliations:** 1 Key Laboratory of Coastal Science and Integrated Management, First Institute of Oceanography, Ministry of Natural Resources of the People’s Republic of China, Qingdao, China; 2 Institute of Marine Development, Ocean University of China, Qingdao, China; Institute for Advanced Sustainability Studies, GERMANY

## Abstract

This study seeks to provide a basic approach to fulfill the spatial visualization of geopolitical environmental factors required for the navigation of vessels in the Arctic. Multi-dimensional geopolitical environmental factors of the Arctic Passage are analyzed and classified into geopolitics, geoeconomics, geo-military, geoculture, and laws and regulations related to geography. Their characteristics are extracted to form an attribute information table matching spatial layers. Based on the information category and basic characteristics, the spatial match method is applied and connected with the spatial layers to examine the spatial point, polyline, and polygon. According to the qualitative description, the study extracted the quantitative indicators for the following spatial–temporal pattern analysis. The standard deviational ellipse is used to analyze the spatial–temporal patterns and trends of the geopolitical environmental indicators of the Arctic Passage in the Arctic and Northeast Asia. The expansion and contraction of geoinformation coexist in the countries surrounding the Arctic Passage. The spatial–temporal changes indicate that the Arctic channel has a great economic impact on the Nordic countries and Northeast Asia, especially the coastal areas of China and Japan. The characteristic extraction and spatial match of the geopolitical environment provide integrated Arctic geoinformation inquiry and services for the diplomatic, administrative, and legal preparations required for Arctic navigation. Therefore, the geospatial analysis conducted provides scientific support and a basis for the geographical distribution and developing trends of visualization and spatial–temporal pattern in Arctic navigation. The results of this research will help decision-makers to make a comprehensive judgment on governance related to the sustainable development of the Arctic Passage.

## 1 Introduction

With the warming of the Arctic Ocean, the ice has been melting at an accelerating speed, making the full opening of the Arctic routes possible [1–5]. Therefore, seeking a suitable method for risk decision-making in Arctic route planning is currently a necessary research topic [6]. The Arctic Passage is closely related to the world geopolitical pattern [7, 8]. The Arctic passages include three routes: the Northern Sea Route (NSR), the Northwest Passage (NWP), and the Transpolar Sea Route (TSR). The environmental and socioeconomic impacts of the Transpolar Sea Route could be locally significant because this route is shorter and deeper than the NSR [9]. The huge benefits generated by the Arctic Passage have gradually attracted the attention of many Arctic countries, which have attached great importance to the Arctic routes and Arctic geopolitics for a long time. Non-Arctic countries, though away from the Arctic, also consider the issue of Arctic routes an important agenda.

During navigation along Arctic routes, ships are not only bound by the maritime administrative regulations of the countries along the route but are also indirectly affected by various factors, such as the political influence, economic development, military, and the culture of each country. So far, services for ship navigation mainly rely on the global positioning system (GPS). Various researchers have conducted a comprehensive analysis of the energy efficiency of ice-going ships to ascertain the most energy-efficient navigation in the Arctic routes for both economic and environmental purposes [10]. A model developed by the researchers estimates how ice conditions change the probability of blockage along the route and play on the economic and environmental attractiveness of the NSR [11].

Some researchers also collected daily information for ships sailing along Russia’s NSR to investigate spatial and temporal variations of shipping and learn about the possible drivers of traffic levels and future trends [12]. In addition, geoinformation support tools have been developed to manage natural risks within the NSR area, which are used to build the geospatial contents and allocate the interconnected components of the solution space [13]. The central Arctic Ocean is designated as a Particularly Sensitive Sea Area under international law, which provides a useful mechanism for creating and updating precautionary shipping measures as more information becomes available [14]. The navigability of passages is affected by many factors, such as meteorological and hydrological conditions, extreme events, facilities, water depth, draft restrictions, and local laws and regulations [15]. The most critical elements restricting Arctic NSR development include extreme climate, political considerations, and sea ice conditions based on expert inputs, whose relations are discussed among these factors as well as their policy implications—as they are likely to contribute to the decision-making of shipping companies and the policy focus of the administration [16].

However, these navigation systems do not provide information on whether the vessels have a legal and political basis for free navigation and scientific investigations in different sea areas. Besides, the related jurisdictional agencies and the economic, political, cultural, and military conditions of the countries, as well as the navigation issues reflected by geoinformation, are not directly concerned. It is against this backdrop that the spatial pattern of the Arctic Passage is analyzed in terms of geopolitical environmental factors, which include regional politics, economics, culture, military, and other factors involved, to achieve the matching of geoinformation with the spatial location and to investigate the spatial–temporal pattern. When planning the Arctic route before navigation and changing the route during navigation, a realistic problem needs to be solved urgently: how to intuitively combine the required geographical environment with spatial information? The findings of this research will play a significant role in aiding the planning of the Arctic navigation routes and facilitating the changes of routes of various countries during the navigation process. At the same time, this paper considers the interaction between the Arctic Passage and its geopolitical and geographical environments. On the one hand, it considers the comprehensive influence of various factors on the Arctic Passage and associates them with geospatial information. From the perspective of spatial visualization, the study analyzes the distribution of the geographical factors of various actors in geographical space and changes the form of word representation of geographical information in the past. Intuitively and comprehensively considering the geographical information of the waterway is beneficial for Arctic navigators when a trip changes before and during the voyage. On the other hand, given the changes in the influence of the geographical environment due to the opening of the Arctic Passage, the study takes the NSR with more navigation as an example, and adopts the spatial analysis method for the spatial–temporal pattern analysis. This step was taken due to the effects of the geopolitical environment in the form of the indicators of multiple influencing factors, which is advantageous to the comprehensive evaluation of the influence of the geographical environment factors of the Arctic Passage.

## 2 Material and methods

### 2.1 Study area

**Fig 1:**
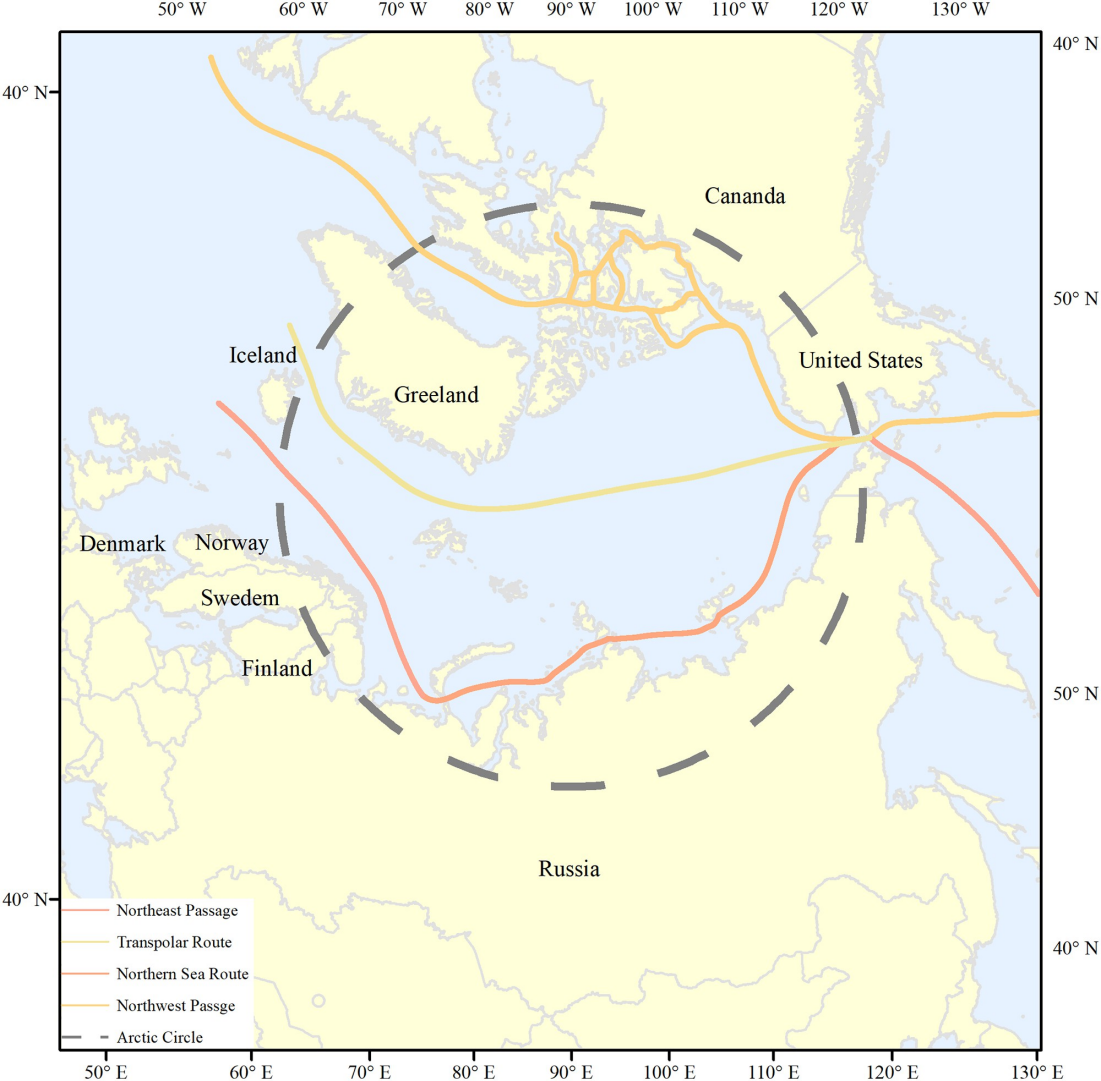
Sketch map of the Arctic Passage. The boundary was obtained from Natural Earth (http://www.naturalearthdata.com/).

The Arctic Passage generally refers to a collection of sea routes across the Arctic Ocean that connect the Pacific Ocean with the Atlantic Ocean. As a result of the seasonal changes in sea ice, Arctic Passage routes are not fixed [17]; instead, there are a number of different routes that mainly consist of the Northeast Channel of the Russian coast [18], the Northwest Channel across the Canadian islands, and the North Pole channel at the center of the Arctic Ocean (Fig 1). The geopolitical environment of the Arctic Passage involves multi-dimensional information about countries or regions during navigation, such as the political, economic, military, cultural, and legal and regulatory issues. Consequently, this paper draws features based on the multi-dimensional geopolitical environment of the Arctic Passage.

### 2.2 Geopolitical environmental factors in the Arctic Passage

#### 2.2.1 Geopolitical factors

The geopolitical factors of the Arctic Passage refer to a type of aggregation between countries in the process of geopolitical right competition in the navigation, i.e., a layout wherein each country is assigned to a certain group. The geopolitical factors surrounding the Arctic waterway are of great importance. On the one hand, they reflect the geopolitical pattern of the Arctic, and on the other hand, they are concerned with the issue of free navigation, which is the focus of global maritime rights [19, 20]. Considering the strong dynamics and extensibility of the Arctic route, its geopolitics should not be the same as those of a fixed area. As a result, the influence of its geopolitics spreads to the extension of the Arctic route [21].

The geopolitical factors of the Arctic Passage not only revolve around the ownership of the seas in the Arctic Ocean but also the terrestrial attributes of coastal countries. Terrestrial attributes are mainly concerned with administrative countries and international organizations with certain Arctic influences, such as Arctic countries with geographical advantages, the Arctic Council, the Arctic Parliament, and the Northern European Council.

#### 2.2.2 Geoeconomic factors

Geoeconomics refers to the economic relationship between countries or regions based on their geographical location, resource endowment, economic structure, etc. The relationship can be an alliance or competition, cooperation, opposition, or even containment [20]. The Arctic Passage has important geoeconomic and commercial value. The economic benefits of the channel are mainly manifested in three aspects. The first is the economic benefit of shipping, i.e., the shipping distance is shortened, which in turn reduces costs. The second is the trade-economic interest; i.e., stakeholders who rely on the Arctic Passage to develop their trade economy have more interest because 90% of their trade is dependent on maritime transportation. The third is the resource and economic interest, which is facilitated by the exploitation of Arctic resources, and this greatly mitigates the energy crisis through the Arctic Passage.

The shipping economy, trade economy, and resource economy in the Arctic region have distinct geographical characteristics, and their economic interests are classified into three categories: countries with exceptional geographical advantages around the Arctic; areas affected by the extension of the Arctic Passage, such as Europe and North America; and the beneficiary countries of the traditional trade route, whose domestic trade economy and energy exports are greatly affected by the opening of the route [21, 22].

#### 2.2.3 Geo-military factors

Geo-military is a combination of the terms “geography” and “military,” and it can be understood as a military situation formed under the influence of geographical factors. The opening of the Arctic Passage has facilitated and diversified military delivery and military operations through the Arctic Ocean, making the region a “new battlefield” for strategic games among the Arctic countries. The Arctic “fights” and the trend of militarization in those countries have intensified. For instance, the United States and Russia attach great importance to military control over the Arctic, and they build military bases, conduct exercises, and deploy anti-aircraft guided missile systems and anti-submarine warfare airplanes in this region. North Atlantic Treaty Organization’s (NATO) eastward expansion and American strategies to fight Russia are likely to exist for a long time, and Russia is also likely to form strategic counter-measures and deterrence against the United States and European countries [23]. However, at present, almost all Arctic countries’ policy statements emphasize that a realistic military threat does not exist in the Arctic. All countries have promised that they will abide by the basic principles of international law to ensure peace and stability in the Arctic.

#### 2.2.4 Factors of laws and regulations related to geography

The utilization of the Arctic Passage must comply with corresponding legal rules. The legal system of Arctic navigation comprises two parts: relevant international law and domestic law. The nature and regulations of the two types of laws are inconsistent. However, as their scopes of application, contents of rules, and legal effects are related to the district to a certain extent, it is necessary to match the laws with geographic information to ensure that the Arctic Passage is used accurately and legally.

#### 2.2.5 Geocultural factors

Geoculture generally analyzes and predicts the strategic situation of the world or a region and the status of cultural expression concerning the political behavior of a country, i.e., according to the geographical form of various geographical factors and political patterns. The existence of geoculture has given birth to different nations and countries, thus influencing relations between countries. In some instances, it has even fueled national war and regional turmoil. The origin of culture, habitat of nationality, and distribution of aborigines in the Arctic are important factors for the investigation of Arctic geopolitical culture. The “cold culture” of the Arctic natives—Eskimos (Inuit)—is also known as the “white culture,” which is largely predominant in the Bering Strait, the Aleutian Islands, Alaska, northern Canada, and Greenland. The Lapps, with their reindeer civilization in the Arctic Nordic region, are Nordic people, Urals, a mix of the Mongolian and European races. They are mainly distributed in the Arctic regions of Norway, Sweden, Finland, and Russia.

### 2.3 Data sources

This paper uses descriptive data, such as interest groups belonging to countries, and quantitative data, such as the Rule of Law Index from the Worldwide Governance Indicators (WGI) project, to measure a country’s geopolitical level. The quantitative data includes GDP, per capita GDP, GDP ratio, and economic density. Therefore, this paper relies on the data provided by the World Bank to measure a country’s geoeconomic level. The spatial point data is used to measure a country’s geo-military level, and it combines the location of the military base with the basic military information. The geocultural level of a country is measured by the employment ratio; the proportion of primary, intermediate, and advanced education; and the Human Development Index (HDI). This data is provided by the World Bank and the United Nations Development Programme. This paper further extracts descriptive data from laws and regulations of various countries and forms an attribute table to explain the laws and regulations.

### 2.4 Spatial match and evaluation methods

#### 2.4.1 Spatial match and evaluation

**Fig 2:**
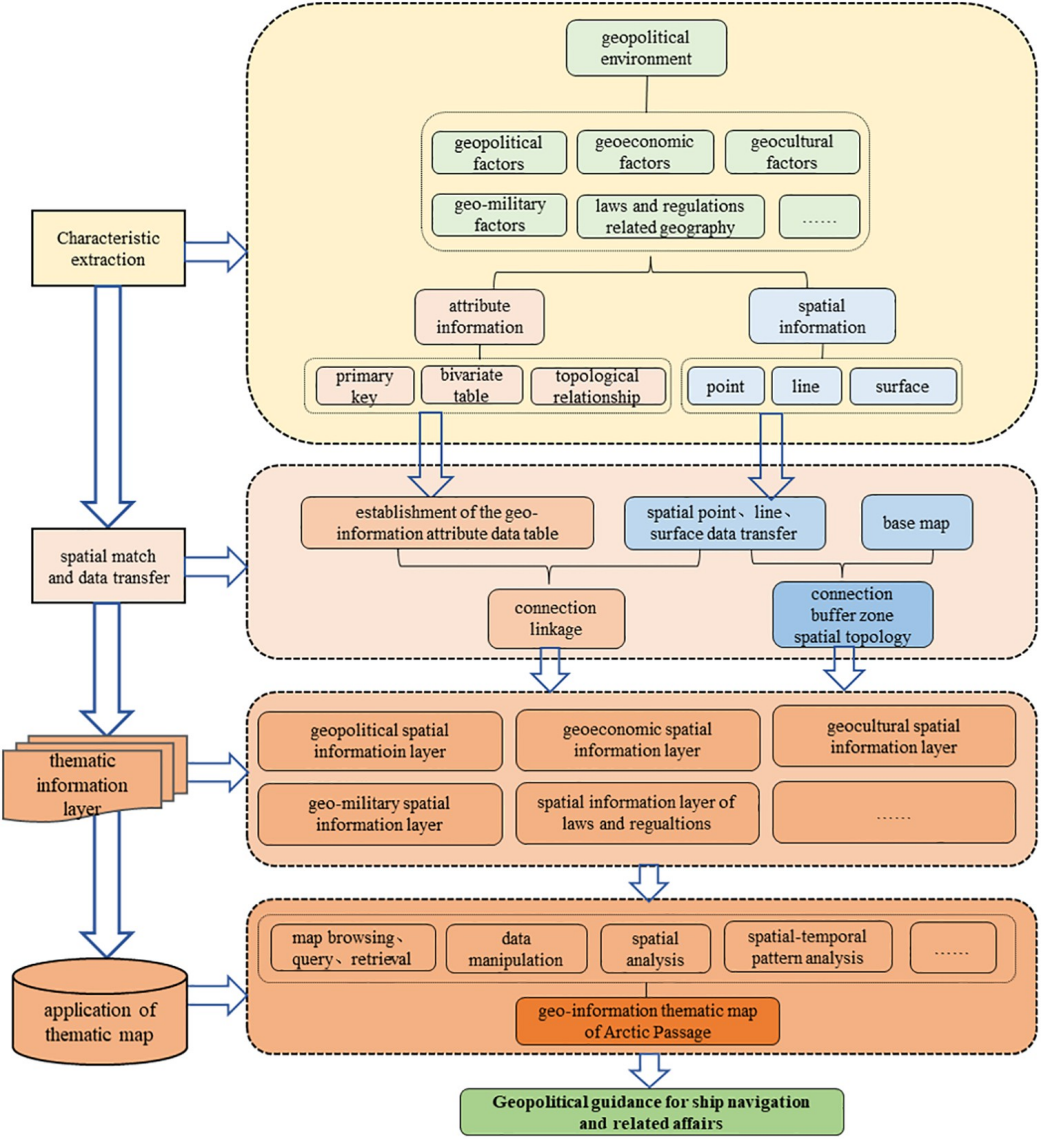
Framework of the spatial match and evaluation of geopolitical environment in Arctic Passage.

Based on the classification of the elements of the geopolitical environment and the analysis of its related influencing factors, this study combines the functions of the Geographic Information System (GIS) to conduct geoinformation matching research in the Arctic Passage [24–27]. The infrastructure and its matching process are determined on the basis of the fundamental logic and ideas of the geopolitical environmental system of the Arctic Passage, as shown in Fig 2. Flexible and simple functions, such as map settings and map analyses of GIS, are employed in the geopolitical environment matching process to provide quantitative and visual geodata support and services for the international activities involving Chinese vessels in the Arctic Ocean.

Matching the geoinformation and geospatial information requires GIS. Supported by computer software and hardware, spatial database, and the theories of systems engineering and information science, the matching process aims to manage and synthesize spatial data as well as data on various topics and provide geoinformation for the planning, decision-making, and management of Arctic activities. Specifically, (1) the attribute of geoinformation is categorized into geopolitics, geoeconomics, geo-military, and geoculture; (2) the spatial match method is used to collect and integrate the multi-dimensional attribute of the geoinformation of the countries involved during the navigation process; (3) according to the properties and characteristics of various geoinformation, relevant attribute tables in the GIS are formed by means of data classification, management, and feature extraction; (4) the basic base map is constructed by matching multi-dimensional attribute information with spatial position—in terms of different types of geography features in the form of points, lines, and surfaces—to obtain a geoinformation layer; and (5) according to various geodata layers and buffer areas within the influence of each segment of the Arctic Passage, the match process provides the necessary thematic map productions with spatial analysis services, as well as the geoinformation browsing based on spatial location, and facilitates the downloading of relevant laws and regulations and a feasibility check of the vessel activities.

The geopolitical environmental spatial match of the Arctic Passage is a kind of “space standardization” based on classification. It not only creates thematic maps according to existing needs but also facilitates basic map browsing, provides space-attribute information, and plays a crucial role in geospatial analysis. This study provides a comprehensive visual information platform for participating in Arctic affairs and meeting the demands of the Arctic scientific research ship in navigation, such as its geographical location and the query of legal provisions. It also highlights the rules and precautions to be observed and the processing procedures of related events during navigation, as well as geoinformation, such as economy, trade, and laws and regulations for the Arctic scientific expedition team. Geospatial analysis can facilitate an independent analysis of points, lines, and surfaces in the relevant areas of the Arctic Passage. It can also achieve a range of statistics and comparative analyses between different areas. Moreover, spatial statistical analysis and spatial analysis can be performed as required. Therefore, this study aims to determine the range and trends affected by the geoinformation of the channel and provide guidelines for Arctic navigation, scientific research, and participation in Arctic affairs to promote resource development and utilization.

#### 2.4.2 Standard deviation ellipse

To ascertain changes in the direction of multiple indicators and measure the spatial distribution of the geographical environment in Arctic countries, which are in close proximity to the Arctic Passage, this paper analyzes the size and centroid of the standard deviational ellipse (SDE) [28]. The SDE describes the spatial distribution characteristics of related elements using the basic parameters of an ellipse, such as the spatial distribution, center, long axis, short axis, and azimuth [29]. The center of the ellipse represents the average center of the spatial distribution of the elements, while the direction of the long axis is the direction of the major trend of the elements. The length reflects the degree of dispersion of the elements in the main trend direction, and the short axis reflects the range of the element’s spatial distribution. It is worth noting that the bigger the axial ratio, the greater the centripetal force of the data is—otherwise, the greater the degree of dispersion is. The azimuth is the angle rotated clockwise from the north direction to the long axis of the ellipse, which represents the direction of the spatial distribution of the elements [30].

## 3 Results and discussion

This paper puts forward three forms of expression related to the analysis and extraction of the elements of the geopolitical environment. The first denotes qualitative descriptive attributes; the second pertains to quantitative index representation; lastly, the third refers to graphical and intuitive expression. First, we analyze and classify various factors that influence geopolitics from a qualitative perspective. Second, we conducted an extraction of characteristics by analyzing the classified elements of the geo-environment and their respective spatial features from the qualitative perspective, and connecting them with spatial match. Moreover, the study found that exploring the indicator representation information from a quantitative perspective according to the classification factors of the geopolitical environment is convenient for data extraction for spatial–temporal pattern analysis.

### 3.1 Characteristic extraction of the geopolitical environment

The study synthesized the factors of the geopolitical environment, including geopolitics, geoeconomics, geoculture, geo-military, and laws and regulations related to geography, as well as extracts that correspond to attributes to establish the link between basic attributes and geographical location. Table 1 reveals that the geopolitical environmental factors of the Arctic Passage feature certain characteristics. The results are presented in the form of layers to illustrate the spatial visualization of multi-dimensional thematic data, such as the spatial match of geodata.

**Table 1:** Basic scheme for geopolitical environmental spatial match.

Thematic data	Characteristics analysis	Realization of spatial match
Geopolitics	(1) Globality in a certain range (2) Regionality with national clusters or administrative divisions (3) Instability due to changes in the current situation	(1) Spatial expression in the form of layers (2) Taking the national administrative division as the basic patch (3) Geopolitical description as the supplementary of the attribute information table (4) Regular or timely updates according to changes in the current situation (5) Evolutionary analysis can be conducted based on previously updated data
Geoeconomics	(1) Regionality with geography as the basic element (2) Administrative divisions with countries as geographic units (3) Spatial and temporal distribution of the economics of geographic units (4) International relevance of the economic development of geographic units	(1) Spatial expression in the form of layers (2) Taking the national and subregional administrative divisions as the basic patch (3) Expression based on the attribute table and taking the index as a field (4) Using attribute table association on the time scale
Geo-military	(1) Regionality with the influence of countries (2) Fixed location of military base (3) Uncertainty of the nuclear submarines under the ice or under the water	(1) Spatial expression in the form of layers (2) Taking the affected area of the country as the fundamental patch (3) Using the military base as the basic point, the area with the detailed data is illustrated using a map to enhance clarity.
Geoculture	(1) Regionality of the national culture (2) Stability and regional continuity in the spatial distribution (3) Characterized with both intercontinental and national distribution	(1) Spatial expression in the form of layers (2) Taking the subregions, such as the county and the province, as the basic patch (3) Taking geo-cultural characteristics as the descriptive attribute data
Laws and regulations related to geography	(1) Uncertainty in geographical location (2) Integrity and continuity of geographical information (3) Spatial–temporal overlap	(1) Spatial expression in the form of spatial layers and attribute tables (2) Taking the buffer zone under the influence of the channel in the article of the laws as the patch (3) Establishing index and linking it with the attribute table

The geopolitical characteristics of the Arctic route can be considered from three dimensions, namely, is the Arctic region, the route and the buffer zone around the route, and the route extending to other areas outside the Arctic Ocean. The Arctic region is a geopolitical combination of five countries in the Arctic, eight countries around the Arctic, and Barents Europe. The scope affected by the route and its buffer zone contains the continental shelf and the exclusive economic zone of such countries. However, the demarcation of the buffer zone may be considered by Arctic countries, such as Russia, Norway, Denmark, and Canada, due to the delimitation disputes in the Arctic. These countries have submitted claims to the Commission on Limits of the Continental Shelf for a continental shelf of 200 nautical miles. Other areas refer to the range where the Arctic Passage can reach outside of the compass of Arctic countries, such as other areas extended by the route. Free navigation is an Arctic geopolitical focus issue that is directly related to the Arctic Passage. Many analyses in geopolitics need to use the quantitative representation of indicators. In this regard, the indicators involving six dimensions of governance in WGI are used, namely, voice and accountability, political stability and awareness of violence/territory, government effectiveness, regulatory quality, rule of law and control of corruption. The indicators correspond to countries to establish a spatial match.

The opening of the Arctic Passage involves not only countries around the Arctic but also countries whose overseas trades are mainly affected by the route. Thus, the study considers all cited countries in terms of geoeconomic factors. Their important ports, foreign trade volume, and resources are factors that need to be considered in geoeconomics. According to the characteristics of geoeconomics in the Arctic Passage (Table 1), spatial match mainly employs economic information as the basic unit and time as the main economic indicator for each country. Table 1 displays the specific match scheme.

The characteristics of geo-military elements reflect the regional influence of the actual military strength of each country. The location of the military bases of the countries is presumed to be in the Arctic, whereas nuclear submarines under the ice or under the water remain uncertain. Among the geo-military factors, the scale of military bases, the number of equipment and personnel, and military expenditure can be extracted as quantitative indicators to extract data for the subsequent evaluation of the geopolitical environment.

The geoculture of the Arctic Passage includes relevant countries and regions of the land and sea areas. It has three main characteristics. First, regionalism is formed mainly in terms of the national culture; second, the stability of spatial distribution depicts regional continuity to a certain extent; third, it possesses the characteristics of intercontinental distribution and national boundaries. The attribute properties are mainly extracted from data on the humanistic environment of the related countries and regions of the Arctic Passage. In addition, the extraction of the humanistic environment index is conducted on the basis of the basic units of the Arctic countries and their subregions. Among the geocultural elements, quantitative indicators can be used to represent the level of education, levels of urban and rural development level, social protection and labor, degree of comprehensive development, and other types of information on the Arctic countries and sub–regions near the Arctic channel. In this manner, index data can be provided for the following analysis of the spatial–temporal evolution of the geopolitical environmental pattern. Table 2 presents the results.

**Table 2:** Arctic geocultural index information.

Category	Factor	Specific index
Education	Level of education	Proportion of primary, intermediate, and advanced education, etc.
Urban and rural development	Population and development indicators	Rural and urban population, grain crops production, food production index, etc.
Degree of comprehensive development	Human Development Index	Human Development Index
	Comprehensive development	Population, education, health, happiness, and cultural diversity
Social protection and labor	Occupation selection	Number of employees in agriculture, industry, service industry, etc.
		Employment ratio, unemployment ratio, female employment ratio, etc.
	Social protection	Social security ratio, female social security ratio, etc.

International law and domestic law form part of the laws and regulations of the Arctic Passage. International law related to Arctic navigation mainly comprises the 1982 United Nations Convention on the Law of the Sea (hereafter referred to as “the Convention”) and the relevant rules of the International Maritime Organization (IMO). First, identifying sea areas with different legal attributes is important to the geographic information of the Arctic Ocean in accordance with the maritime delimitation rules established by the Convention. Second, navigation rights vary in seas with different legal attributes, such as from internal waters and the territorial sea to the exclusive economic zone, the high seas, and the “straits used for international navigation.” Therefore, we need to identify corresponding navigation rights with different legal attributes. Third, general navigation rules and the special rules of the IMO on polar navigation are extremely detailed and complex. However, they lack a distinction on the legal attributes of the sea. Among these rules, four convention systems, namely, the International Convention for the Safety of Life at Sea, the 1973/1978 International Convention for the Prevention of Pollution from Ships and its Protocol in 1978 (MARPOL 73/78), the International Convention on Standards of Training, Certification and Watchkeeping for Seafarers (STCW), and the International Guidelines for the Navigation of Ships in Polar Waters, adopted in 2014 [31] (Polar Code) (Polar Rules for short), are the main rules. The characteristics of these international laws are extracted according to the degree of correlation with geographical location, that is, continuous but with necessary discontinuity. Table 3 provides the interpretation of certain articles and feature extractions. The quantitative index of laws and regulatory geography can consider the strength of the legal rights of countries adjacent to the Arctic Passage.

**Table 3:** Characteristics of laws and regulations in the Arctic Passage.

Category	Laws and regulations	Characteristics
International law	*United Nations Convention on the Law of the Sea*	Navigation rights vary in the seas with different legal attributes, from the internal water, territorial sea, exclusive economic zone, and high seas, to the “straits used for international navigation”
	*International Guidelines for the Navigation of Ships in Polar Waters* (Polar Code)	Stricter standards of environmental protection than MARPOL 73/78 are adopted, requiring ships operating in specific Arctic waters to apply for polar ship certificates ……
	*International Convention for the Prevention of Pollution from Ships and its Protocol* (MARPOL 73/78)	
	*International Convention for the Safety of Life at Sea*	
	……	
Domestic law	Domestic law of Russia	Relevant regulations of the Northeast Passage concerning the geographical location: (1) The waters adjacent to the northern coast of the Russian Federation consist of internal waters, territorial seas, contiguous areas, and exclusive economic zones; ……
	Domestic law of Canada	Relevant regulations of the Northwest Passage concerning the geographical location: (1) 60 degrees north latitude and 141 degrees west longitude in Canadian territorial waters and exclusive economic zones; (2) the boundaries of the exclusive economic zone between Canada, Greenland, and Denmark; ……
	……	……

### 3.2 Spatial match of the geopolitical environment

In this study, spatial match mainly involves geospatial data and attribute data as well as necessary base map data. Spatial data consists of three forms of data: point, polyline, and polygon. It is generally constructed from point to line and then to surface. Attribute data is geoinformation characteristic data, and it includes geopolitics, geoeconomics, geoculture, geo-military, and laws and regulations. As the base map data covers much more information, it is managed by establishing a base map vector database, which mainly includes administrative divisions, Arctic stations, coastlines, ice boundaries, rivers, and Arctic Ocean ranges.

According to the information of the Arctic scientific research station and the important marks of the coastal countries, the corresponding point-like vector data are formed using the drawing tool on a desktop, and it is based on the information about the baseline and the external boundary of the territorial sea, the relevant connection rules and relationships, and the point-like vector data that are transferred into linear data. In terms of the different definitions of different sea areas and exclusive economic zones depending on the countries concerned and the boundaries of each region, relevant surface data are obtained. To ensure consistency of data projection, all the thematic data and base map data are projected using Polar Stereographic Projection with a standard latitude of 71°N.

#### 3.2.1 Spatial match of the geopolitics

**Fig 3:**
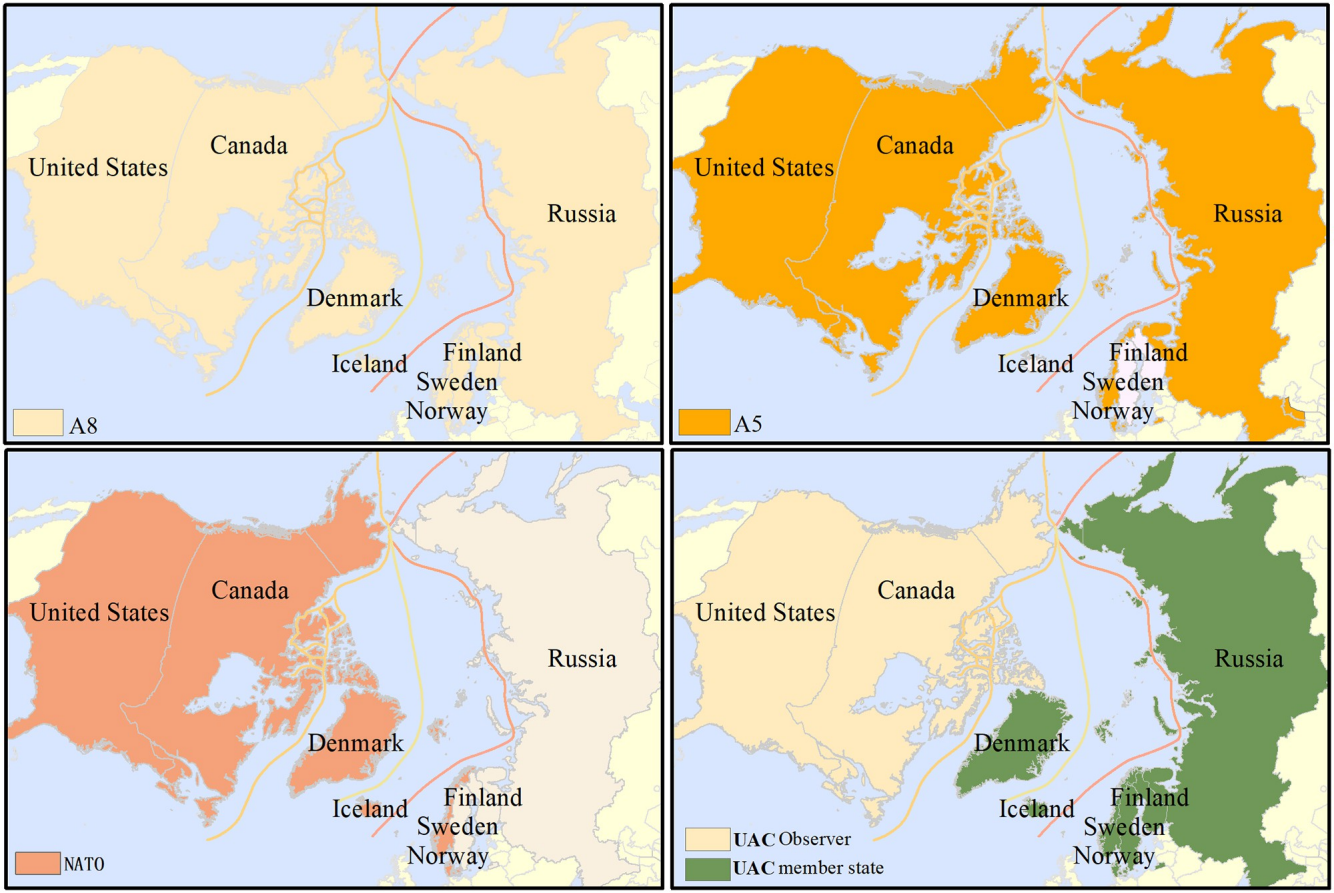
Four examples of geopolitical characteristic extraction and spatial match. The boundary was obtained from Natural Earth (http://www.naturalearthdata.com/).

According to the extraction of characteristics and its relationship with space, in terms of the geopolitical information of the Arctic passage, the relationship between the countries or groups of countries involved must be viewed globally, and the geographical information of the political pattern should be interpreted from a global or large regional perspective. Based on the classification of attributes, the geopolitical plate is determined by different classifications of geographical attributes, which mainly include the geopolitical patches in accordance with the interest groups of the Arctic Passage and the interest groups involved in Arctic affairs. When considering geopolitical factors, the state is the basic unit of the terrestrial factors, which can be analyzed based on three major interest groups: the Arctic eight countries (A8), the five coastal countries (A5), and the extraterritorial countries (Fig 3). Some geopolitical characteristics are shown in Table 4, which illustrates the present identity of each country in different international organizations, including whether it has joined the organization and its specific status. The issue of the delimitation of the continental shelf revolves around the ownership of the sea area, and it is mainly based on the documents approved by the Commission on Limits of the Continental Shelf. In this context, delimitation also refers to the Arctic countries’ application program for the continental shelf of the Arctic Ocean, which includes the territorial sea, the adjacent area, the exclusive economic zone, and the continental shelf.

**Table 4:** Geopolitical features of the Arctic Passage.

Country Name	Interest group of the channel	Arctic Council	Barents Europe	Northern European Council	Arctic University Alliance	European Union membership	Arctic Scientific Committee	European Arctic Commission	Pacific Arctic Organization	NATO member states
Canada	A5, A8	Presidency	Observer of European Council		Yes		Yes	Observer	Yes	Yes
Russia	A5, A8	Presidency	Yes				Yes	Member state	Yes	
Iceland	A8	Member state	Yes	Yes	Yes			Member state		Yes
Finland	A8	Member state	Yes	Yes	Yes	Yes	Yes	Member state		
Sweden	A8	Member state	Yes	Yes	Yes	Yes	Yes	Member state		
The United States	A5, A8	Member state			Yes		Yes	Observer	Yes	Yes
Denmark	A5, A8	Member state	Yes	Yes	Yes	Yes		Member state		Yes
Norway	A5, A8	Member state	Yes	Yes	Yes			Member state		Yes
China	Extraterritorial country	Observer			Yes		Yes		Yes	
Japan	Extraterritorial country	Observer			Yes		Yes	Observer	Yes	
……										

#### 3.2.2 Spatial match of the geoeconomics

**Fig 4:**
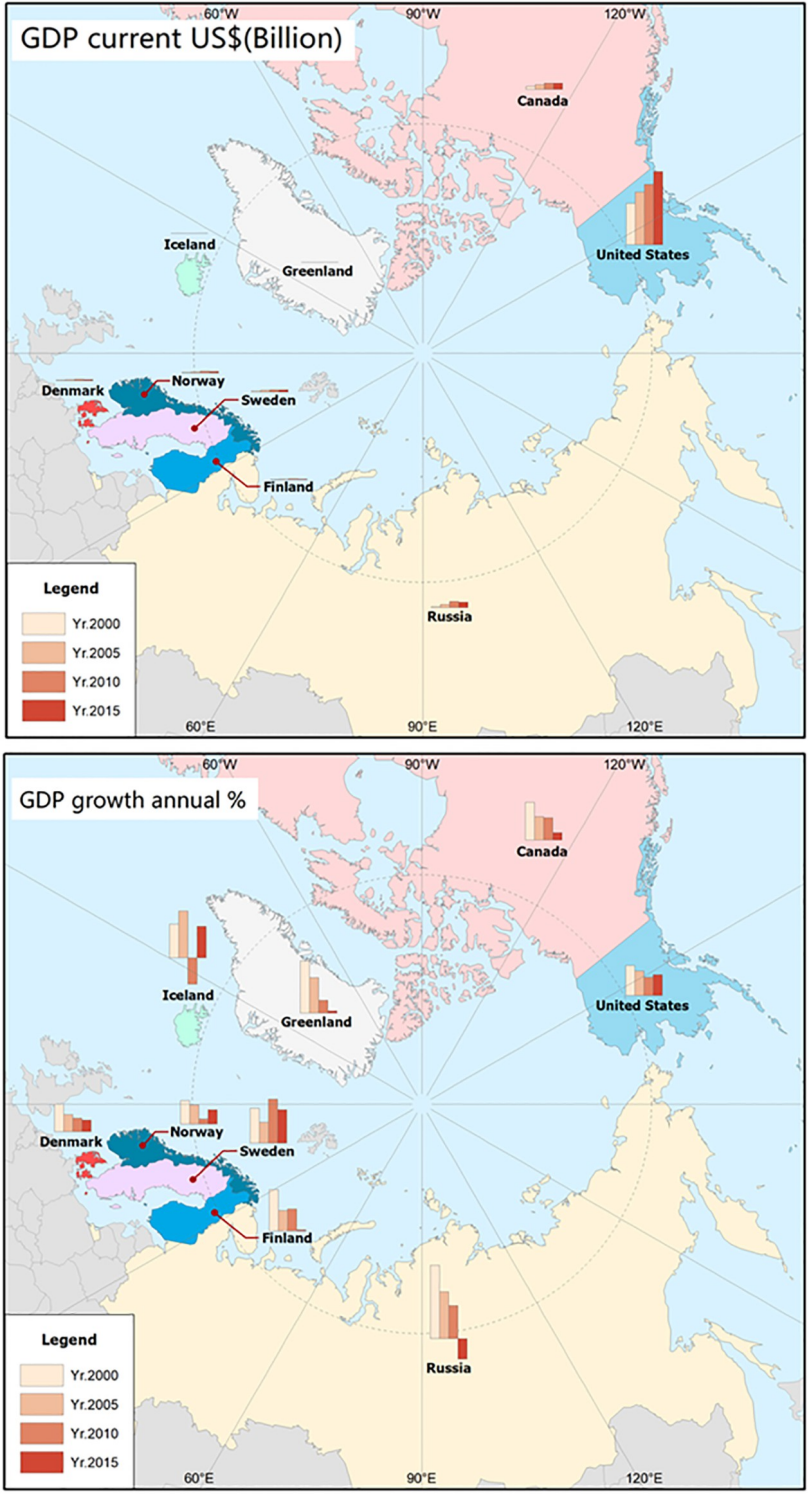
Geoeconomic characteristic spatial match, taking GDP and GDP growth as examples. The boundary was obtained from Natural Earth (http://www.naturalearthdata.com/).

The actual geographical location, geoeconomic factors related to ports, and the number and tonnage of vessels are mainly united in the form of point, polyline, and polygon to achieve spatial expression in the form of patches. When solving the problems of international correlation, spatial analysis is adopted with economic indicators as the mainline. Therefore, the geoeconomics of the Arctic Passage is characterized by the nation and other subregions, such as counties and provinces, as the basic units. After collecting various economic data and extracting the characteristics, the specific features and index information were recorded in the attribute table (Table 5). The index data were also matched with the spatial map, and GDP growth was used as an example (Fig 4).

**Table 5:** Geoeconomic characteristics and index information table.

Region	Country	Subregion	Index
**Northern Europe**	Norway	Finnmark Fylke	It mainly includes population, economy, labor force, health, education, and social services. (1) Economic data: GDP, per capita GDP, GDP ratio and economic density (2) Industrial structure: proportion of primary, secondary, tertiary, and specific industries (3) Land data: land area and proportion of land area ……
		Nordland Fylke	
		Svalbard	
		Troms Fylke	
	Sweden	Norrbottens Län	
		Västerbotten Län	
	Finland	Lapland	
		Kainuu, Oulun Lääni	
		Northern Ostrobothnia	
	Iceland		
	Denmark		
**Russia**	Russia	Republic of Karelia	
		Murmansk	
		Alhamagelsk	
		Nenetskiy Avtonomnyy Okrug	
		Komi Republic	
		Yamalo-Nenetskiy Avtonomnyy Okrug	
		Krasnoyarskiy Kray	
		Chukotka Autonomous Okrug	
**North America**	Canada	Yukon Territory	
		Northwest Territories	
		Nunavut Territory	
	Alaska		
	Greenland		
**Other areas**	……		

#### 3.2.3 Spatial match of geo-military

**Fig 5:**
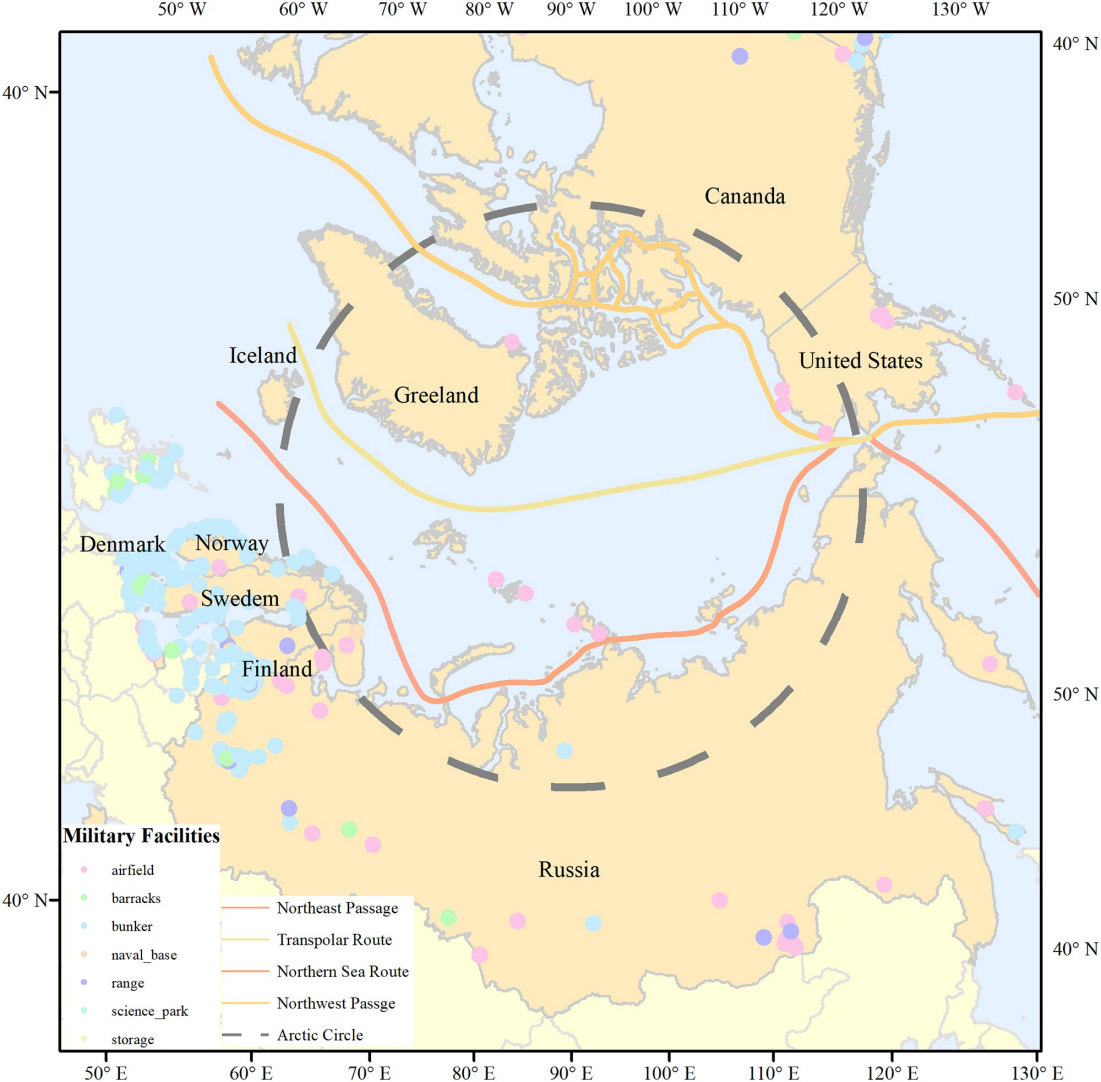
Example of the point of geo-military characteristic spatial match. The boundary was obtained from Natural Earth (http://www.naturalearthdata.com/).

Therefore, this paper organizes the basic military information mainly from two angles: the spot (Fig 5) and the entire area. First, it combines the location of the military base with the basic military information, and second, it focuses on the military forces of various countries and the regional influence of each country. Meanwhile, the geo-military information is mainly based on the spatial expression of the buffer zone under the Arctic countries’ military influence, which combines the space and attributes with the military-based location data as the mainline. Simultaneously, the influence of different patches’ information on ice and sea is considered.

#### 3.2.4 Spatial match of geoculture

According to the geocultural structure of the Arctic, the area is divided in terms of the location of geographical and cultural distribution. Analysis of the geo-cultural information of different countries and regions can help the fleet effectively avoid various ethnic contradictions during the Arctic navigation and to facilitate navigation activities. In the Arctic, or the circumpolar region, the people are indigenous inhabitants of the northernmost regions of the world; therefore, they can represent geoculture to a certain extent. For the most part, they live beyond the climatic limits of agriculture, where climatic gradients determine the effective boundaries of the circumpolar region. Geocultural spatial match is mainly based on the buffer zone in the subregion, ethnic distribution, and the spatial analysis of cultural characteristics and international relations.

#### 3.2.5 Spatial match of Laws and regulations related to geography

All coastal countries have domestic laws concerning Arctic navigation and the laws are applicable within the sea areas under their jurisdiction. Although these laws should be based on the international conventions that specific countries have ratified, differences in geographical location, national strength, and foreign policy have led to the selection of different governance models at the domestic law level [32, 33]. For example, because of geographical advantages and historical reasons, Russia and Canada have a strong control tendency over the Northeast Passage and the NWP. The governance of the channel is mainly based on the ambiguity principle of the United Nations Convention on the Law of the Sea that allows the expansion of power through domestic legislation. On the contrary, some countries, such as Norway and Iceland, manage the channel by formulating specific implementation regulations based on the relevant international conventions that they have ratified. Information on such domestic law was interpreted, and the feature extraction was carried out according to the description of the geographical location in the article. Where necessary, the segmentation features of the channel were extracted according to the provisions of laws and regulations, and the data influenced by the laws and regulations were extracted by surface features.

**Fig 6:**
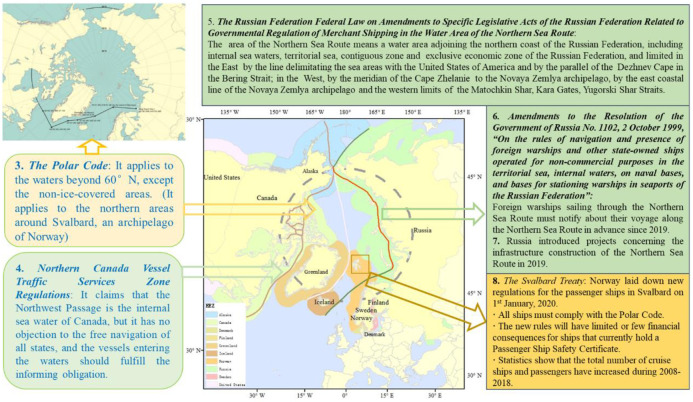
Example of laws and regulations spatial match. The boundary was obtained from Natural Earth (http://www.naturalearthdata.com/). https://www.britannica.com/place/Arctic/The-people.

The two types of laws (i.e., international law and domestic law) need an interpretation that matches their geographical location, can extract relevant characteristics, form attribute lists, and coordinate with geographical positions. In terms of the characteristics of laws and regulations of the Arctic Passage, considering all the factors above and the consistency of attribute and spatial information, this paper interprets the laws and regulations and forms an attribute table. Fig 6 depicts a case of map representation as an example. Based on the specified provisions, the Arctic Passage is segmented, and the range of the buffer zone is determined. The overlapping areas can be illustrated with different layers.

### 3.3 Spatial–temporal pattern analysis

According to the multi-dimensional data of the Arctic Passage obtained above, the spatial pattern is divided. This study aims to analyze the geopolitics, geoeconomics, and geoculture patterns of the target areas. This paper mainly takes the country as the basic unit, selects corresponding indicators from geopolitics, geoeconomics, geo-military, geoculture, and legal and regulatory efficiency, and performs spatial statistical analysis based on the indicators. Corresponding thematic maps are produced and used as needed. The thematic maps contain a large amount of information in the form of images, symbols, annotations, and colors, which can provide basic geodata for polar scientific research. In addition, this paper clearly represents the temporal and spatial distribution features of various cartographic objects and the connection among them.

#### 3.3.1 Analysis of spatial patterns based on SDE

**Fig 7:**
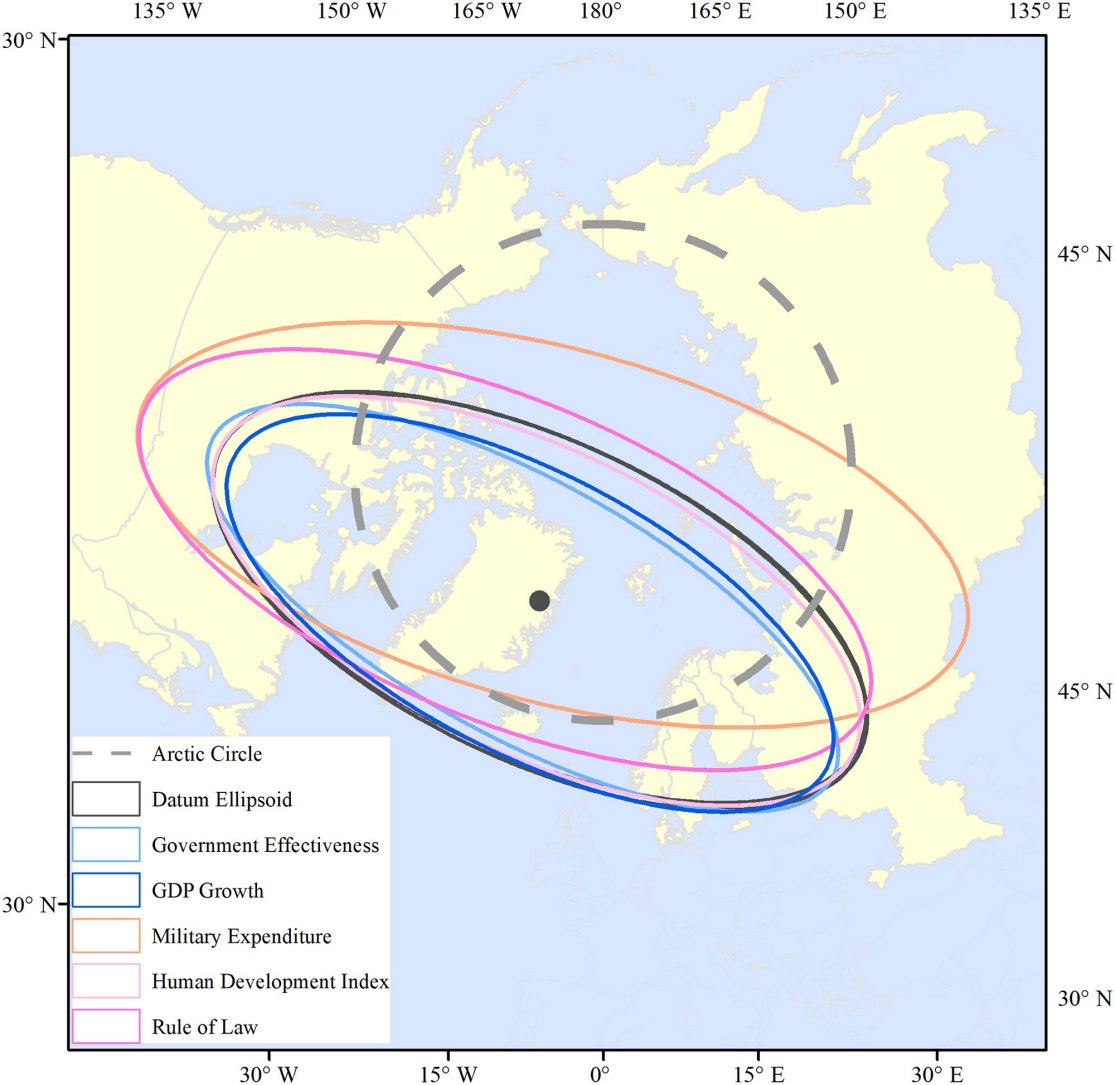
SDE of related geopolitical environmental indicators in the Arctic Passage. The boundary was obtained from Natural Earth (http://www.naturalearthdata.com/).

According to the classification of geopolitical environmental information and the characteristics of the index, government effectiveness, GDP growth, military expenditure, HDI, and rule of law were selected to represent different types (S1 Table). Based on the SDE of the respective index, the results are shown in Table 6 and Fig 7. As for the government effectiveness, both the SDE axis and area are smaller than the datum ellipsoid, which is a clear indication that the index is shrinking in Arctic countries. The coordinates of the center move to the southwest and the azimuth gets larger, implying that the government effectiveness of the Nordic countries has a positive and strong effect on stimulating the Arctic. The GDP growth represents geoeconomics, and the overall spatial distribution pattern shrinks in the Nordic countries. Apparently, the proportion of military expenditure increases greatly and the center moves to the northwest, expanding toward Russia, Canada, and the United States. The expansion trend is quite obvious, indicating that the military forces of Russia and the United States have a stronger spatial layout in the Arctic region, which is also consistent with the density of military base stations. According to the distribution pattern of the HDI, the SDE and the datum ellipsoid have a high degree of consistency. Notably, the focus is slightly inclined to the southwest, and the spatial layout is slightly contracted toward the United States and Canada. The SDE of the Rule of Law expands to the northwest, but the degree of expansion is less than that of military expenditure. This is closely related to the fact that Russia controls the Northeast Channel through its domestic law and Canada controls the Northwest Channel.

**Table 6:** Spatial layout parameters of geopolitical environmental information in the Arctic.

Parameters of Ellipse	Area/km^2^	Long Axis/km	Short Axis/km	Axial Ratio	Azimuth
Datum Ellipsoid	200186512.016	3773.209	1688.972	2.234	115.275
Government Effectiveness	163426320.947	3782.735	1375.420	2.750	119.389
GDP Growth	168733990.157	3585.947	1497.797	2.394	117.722
Military Expenditure	271623209.304	4574.179	1890.424	2.420	105.321
Human Development Index	193186609.969	3763.744	1634.022	2.303	116.230
Rule of Law	225962251.439	4183.335	1719.609	2.433	112.933

#### 3.3.2 Analysis of spatial–temporal evolution based on SDE

**Fig 8:**
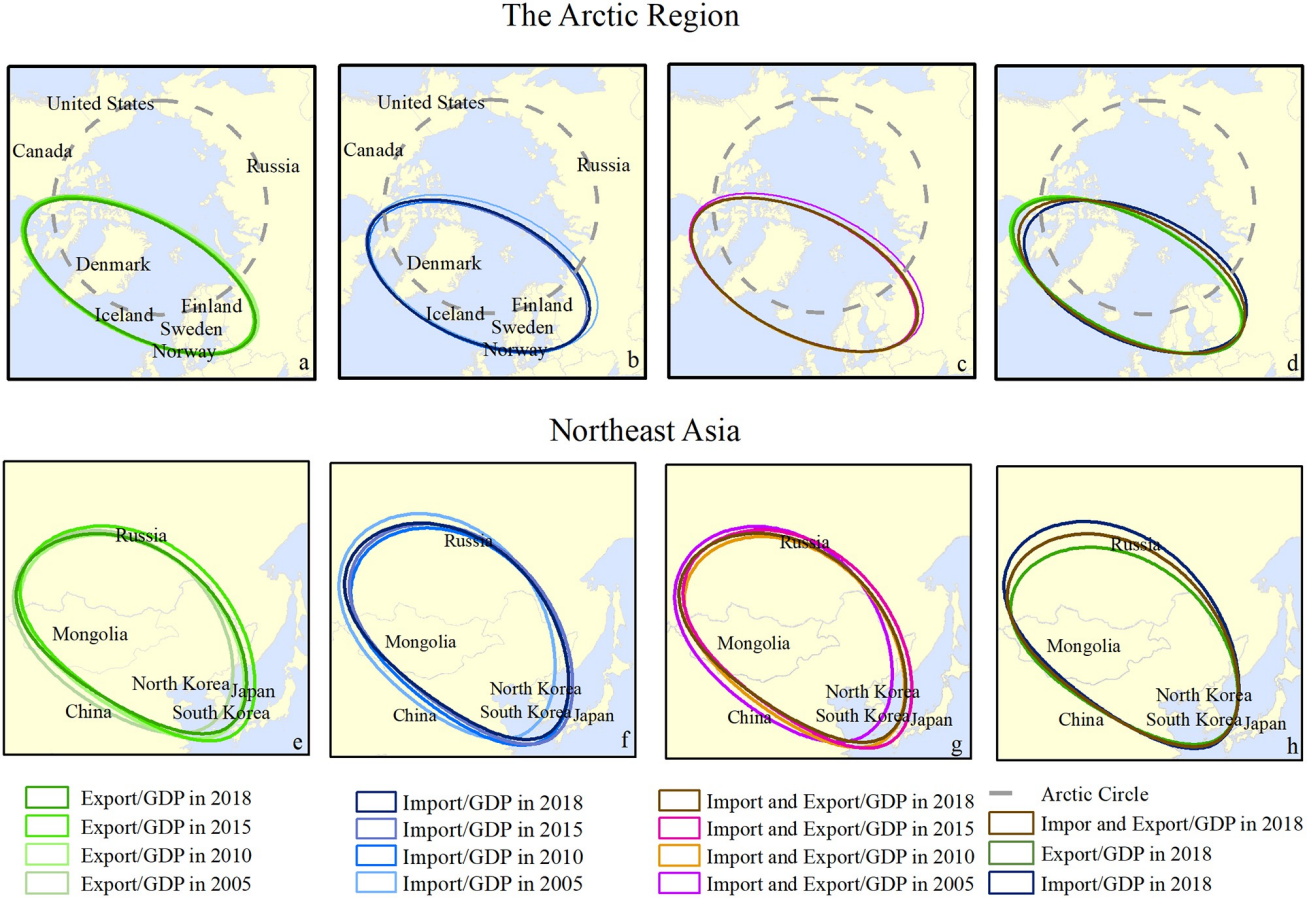
Movement of the standard deviational ellipse and centroid of foreign trade indicators in the Arctic region and Northeast Asia. The boundary was obtained from Natural Earth (http://www.naturalearthdata.com/).

The distribution pattern of import and export indicators in the Arctic region (S2 Table) shows an evolution pattern from west to east and from south to north—that is, a pattern of “west (slightly north) and east (slightly south)”—as shown in Fig 8. According to the SDE, from 2005 to 2018, the distribution range of export GDP reduced from 15 175 261. 806 km^2^ to 14 113 814. 958 km^2^, which shows a trend of spatial contraction, as illustrated in Fig 8a. The coordinates of the SDE centroid have been changed from (22.234W, 73.266N) to (21.783W, 72.785N). The azimuth gets larger and the long axis of the SDE becomes shorter, which is a clear indication that the main contraction force driving the Arctic export volume is east–west, rather than north–south. This also reflects a reduction in Russia and Canada’s export as a result of the contraction of the ellipse. According to Fig 8b, the SDE of Arctic countries’ import GDP contracted to the southwest from 2005 to 2015. During this period, it remained relatively stable from 2010 to 2015, although it contracted slightly to the northwest. From 2015 to 2018, it slightly expanded toward the northeast, and the extended area is 236303.154km^2^. Overall, from 2005 to 2018, the SDE tended to contract toward the southwest. During the period 2005–2018, especially 2010–2015, the SDE of the import and export GDP contracted to the southwest, as shown in Fig 8c. Comparing the SDE of the import GDP and the export GDP in 2018, as presented in Fig 8d, the long axis of export volume is longer while the short axis is shorter than that of the import volume. The export volume stretches in the east–west direction but contracts toward the north–south direction, which is a clear indication that the export volume extends to the Nordic countries, Canada, and the United States. The general trend shows that with the opening of the Arctic Passage, the spatial distribution of Arctic countries’ international trade as a share of the GDP is contracting toward the Nordic countries, Canada, and the United States. This implies that the Arctic Passage has increased the proportion of foreign trade in Nordic countries’ national economies.

According to the SDE of the spatial distribution of imports and exports in Northeast Asia, the distribution pattern is characterized by the northwest to the southeast direction, as shown in Fig 8. The SDE of export GDP shows a state of expansion and a subsequent contraction, as shown in Fig 8e, where the azimuth gets smaller and the general direction approaches southeast. The spatial distribution of import GDP in the Northeast Asia countries shows a tendency toward the southeast, as shown in Fig 8f. This manifests a trend of expansion from 2005 to 2015, i.e., as the size of the long axis of the ellipse becomes larger, the short axis becomes shorter and the oblateness gradually increases. A slight contraction was observed in 2018, but the azimuth continues getting smaller and the overall trend toward the southeast is maintained. From 2005 to 2015, the spatial distribution of import and export GDP also expands to the southeast, as shown in Fig 8g. It remains in a state of contraction from 2015 to 2018 as the azimuth gets smaller and the oblateness gradually increases. As shown in Fig 8h, the export GDP is slightly smaller than the import GDP, but it tends to approach China, Japan, and South Korea. The fact that the contribution of foreign trade to the national economy for Northeast Asian countries tends to expand toward the southeast indicates that in recent years, with the opening of the Arctic Passage, the export volume of the countries in Northeast Asia has played a significant role in their economic growth, especially in China and Japan.

Therefore, after the analysis and discussion, spatial match can be conducted on the basis of the relationship between feature extraction and geographic information to establish a spatial association, which is represented by spatial data. The innovation of establishing a connection between geopolitical analysis and spatial mapping is to utilize the geopolitical environmental methodology that integrates qualitative analysis, quantitative characterization and spatial match. The practicability of this approach is that it can not only render the geopolitical environment more intuitive and visual but also promote in-depth analysis of the spatial–temporal evolution. Moreover, it provides effective research ideas and tools for researchers, stakeholders and decision makers of the Arctic Passage in analyzing problems from the perspective of space, time change, and volume difference.

## 4 Conclusion

The geopolitical environment of the Arctic Passage not only refers to geography and politics, but also consists of many humanistic societies and their influencing factors related to geography. Based on the high demand for the geospatial information about the Arctic Passage in navigation and scientific research activities, this paper investigates geoinformation match methods, where the results provide a geopolitical environmental methodology that integrates qualitative analysis, quantitative characterization and spatial match of Arctic Passage, which not only renders the geopolitical environment more intuitive and visual, but also promotes in-depth analysis of spatial–temporal evolution. In practical application, on the one hand, it provides integrated Arctic geoinformation query and retrieval services for Arctic expeditions and commercial navigation to ensure access to important information that must be known and observed in the process of course changes, such as geopolitics, geoeconomics, and laws and regulations, which play a significant role in the diplomatic, administrative, and legal aspects of the Arctic navigation. On the other hand, it provides visual, demand-oriented, and spatial–temporal geodata support and services required to ensure efficiency in the international affairs of vessels in the Arctic Ocean. Third, an analysis of spatial statistics was conducted on the basis of the indicators of geoinformation, geographical spatial distribution, and the spatial–temporal pattern of the Arctic Passage using SDE.

Therefore, this study aimed at achieving three objectives: (1) Analyzing the geoinformation of the Arctic Passage and classifying it into different perspectives, including geopolitics, geoeconomics, geo-military, and geoculture. The characteristics are extracted to form relevant attribute tables with the GIS tools. The information related to the geographical location is also extracted to connect with the attributes. (2) Determining the spatial match method based on the information category and attribute characteristics, according to the application of geoinformation in the form of point, line, and surface. The attribute information concerning the Arctic navigation channel, such as economics, politics, culture, military, and laws and regulations, is linked and matched with the spatial information to provide a basis for conducting the spatial analysis of the geopolitical environmental information of the Arctic Passage. (3) According to the qualitative description of the geopolitical environmental factors of the Arctic Passage, the study extracted the quantifiable indicators for quantitative analysis to establish the relationship with geospatial mapping. (4) Realizing spatial statistical analysis in terms of the specific research project and its requirements. The extraction of indicators is based on the related elements in the geoinformation of the Arctic Passage, such as geopolitics, geoeconomics, geo-military, geoculture, and laws and regulations, and the application demand is to perform an SDE spatial statistical analysis toward the Arctic and Northeast Asia. According to the geoinformation of the Arctic, military expenditure and Strength of Legal Rights Index stretch toward Russia and Canada, while government effectiveness, GDP growth, and HDI contract toward Nordic countries, the United States, and Canada. Based on the analysis of the foreign trade index of the Arctic and Northeast Asia, the opening of the Arctic Passage plays a significant role in motivating the economy of countries in Northern Europe and Northeast Asia, especially in China and Japan. This research is helpful for those who care about the Arctic Passage and for decision-makers in making a comprehensive judgment on governance related to the sustainable development of the navigation channels in the Arctic.

## Supporting information

S1 Table Dataset records of representative indicators in geopolitical environment of the Arctic Passage used for spatial pattern analysis. (XLSX)

S2 Table Dataset records of the import and export indicators were used to assess the spatial-temporal evolution. (XLSX)

## References

[1] Khon VC, Mokhov II, Latif M, Semenov VA, Park W. Perspectives of Northern Sea Route and Northwest Passage in the twenty-first century. Climatic Change. 2010;100:757–768. 10.1007/s10584-009-9683-2.

[2] Chen JL, Kang SC, Meng XH, You QL. Assessments of the Arctic amplification and the changes in the Arctic sea surface. Advances in. Climate Change Research. 2020;10(4):193–202. 10.1016/j.accre.2020.03.002

[3] Wang CY, Yang YD, Zhang J, Tian B, Ding M. Research on sea ice variability and navigation of the Arctic Northwest Passage from remote sensing data. Chinese Journal of Polar Research. 2020;32(2): 236–249. 10.13679/j.jdyj.20190043.

[4] Cyl A, Hmf A, Xjd B, et al. The Arctic policy and port development along the Northern Sea Route: Evidence from Russia’s Arctic strategy. Ocean & Coastal Management. 2020;201. 10.1016/j.ocecoaman.2020.105422.

[5] Gunnarsson B. Recent ship traffic and developing shipping trends on the Northern Sea Route—Policy implications for future arctic shipping. Marine Policy. 2021;124. 10.1016/j.marpol.2020.104369.

[6] Li Z, Hu SP, Gao GP, Yao CY, Fu SS, Xi YT. Decision-making on process risk of Arctic route for LNG carrier via dynamic Bayesian network modeling. Journal of Loss Prevention in the Process Industries. 2021;71. 10.1016/j.jlp.2021.104473.

[7] Katysheva EG. The role of the Northern Sea Route in Russian LNG projects development. IOP Conference Series: Earth Environmental Science. 2018;180. 10.1088/1755-1315/180/1/012008.

[8] Verny J, Grigentin C. Container shipping on the Northern Sea route. International Journal of Production Economics. 2009;122(1):107–117. 10.1016/j.ijpe.2009.03.018.

[9] Bennett MM, Stephenson SR, Yang K, Bravo MT, Jonghe BD. The opening of the Transpolar Sea Route: Logistical, geopolitical, environmental, and socioeconomic impacts. Marine Policy. 2020;121. 10.1016/j.marpol.2020.104178.

[10] Zhang C, Zhang D, Zhang MY, Mao WG. Data-driven ship energy efficiency analysis and optimization model for route planning in ice-covered Arctic waters. Ocean Engineering. 2019;186. 10.1016/j.oceaneng.2019.05.053.

[11] Cheaitou A, Faury O, Cariou P, Hamdan S, Fabbri G. Economic and environmental impacts of Arctic shipping: A probabilistic approach. Transportation Research Part D: Transport and Environment. 2020;89. 10.1016/j.trd.2020.102606

[12] Li XY, Otsuka N, Brigham LW. Spatial and temporal variations of recent shipping along the Northern Sea Route. Polar Science. 2020;27(12). 10.1016/j.polar.2020.100569.

[13] Abramov V, Popov N, Shilin M. Geo-information support tools for natural risks management within Northern Sea Route. Transportation Research Procedia. 2021;54:144–149. 10.1016/j.trpro.2021.02.058.

[14] Stevenson TC, Davies J, Huntington HP, Sheard W. An examination of trans-Arctic vessel routing in the Central Arctic Ocean. Marine Policy. 2019;100:83–89. 10.1016/j.marpol.2018.11.031.

[15] Stephenson SR, Smith LC, Brigham LW, Agnew JA. Projected 21^st^ century changes to Arctic marine access. Climatic Change 2013;118(3):885–899. 10.1007/s10584-012-0685-0.

[16] Wan Z, Nie A, Chen JH, Ge JW, Zhang C, Zhang Q. Key barriers to the commercial use of the Northern Sea Route: View from China with a fuzzy DEMATEL approach. Ocean & Coastal Management. 2021;208. 10.1016/j.ocecoaman.2021.105630.

[17] Frank SO. Opening speech: International shipping on the Northern Sea Route—Russia’s perspective. Proceedings of the Northern Sea Route User Conference. 1999, Nov 18–20; Oslo. Dordrecht: Springer, 2000. 10.1007/978-94-017-3228-4_3.

[18] Wang L, Zhao Y, Liu JM, Han SQ. China’s first trans-arctic voyage and related expectations. Chinese Journal of Polar Research (in Chinese). 2014;26(2):276–284. 10.13679/j.jdyj.2014.2.276.

[19] Lang YH, Wang LM. Evolution of petroleum geopolitical patterns and China’s policy response. Resources Science (in Chinese). 2008;12(30):1778–1783. https://doi.org/CNKI:SUN:ZRZY.0.2008-12-001.

[20] Guo NR, Hu MX. Evaluation of comprehensive strength of stakeholders in the Arctic passage based on geo-economic benefits. Marine Economy (in Chinese). 2018;6(8):3–12. 10.19426/j.cnki.cn12-1424/p.2018.06.001.

[21] Zhang X, Yang HG, Wang L. Strategic thinking on China’s involvement in the development of Arctic sea route. Chinese Journal of Polar Research (in Chinese). 2016;2(28):267–276. 10.13679/j.jdyj.2016.2.267.

[22] Li ZF, Li WY. Evolution of political framework of Arctic political hinterland. Journal of Tonghua Normal University (in Chinese). 2018;2(39):41–49. 10.13877/j.cnki.cn22-1284.2018.03.007.

[23] Tang Y. An analysis of the new trend of remilitarization in the Arctic regions and its characteristics. Journal of Jiangnan Social University (in Chinese). 2015;2(17):44–49. 10.16147/j.cnki.32-1569/c.2015.02.009.

[24] Yang Y, Xiong B, Peng S, Liu S, Chen H, Zhang T. Transient electromagnetic characteristics of coal seams intruded by magmatic rocks. PLOS ONE. 2022;17(2): e0263293. 10.1371/journal.pone.0263293 35171937 PMC8849541

[25] Chen FB, Zhu J, Wang JB, Yang Y, Iqbal I, Zhang TY, Li P. Seismic attribute analysis of coal seams intruded by magmatic rock. Petroleum Science and Technology. 2022. 10.1080/10916466.2022.2033263.

[26] Zhu J, Jin S, Yang Y, Zhang TY. Geothermal resource exploration in magmatic rock areas using a comprehensive geophysical method. Geofluids. 2022. 10.1155/2022/5929324.

[27] Yang Y, Xiong B, Peng SX, Chen HB, Zhang TY, Liu L. Geothermal exploration using numerical simulation and a comprehensive electromagnetic method. Petroleum Science and Technology. 2022. 10.1080/10916466.2022.2060256.

[28] Li DR, Yu HR, Li X. The spatial-temporal pattern analysis of city development in countries along the Belt and Road Initiative based on nighttime light data. Geomatics and Information Science of Wuhan University (in Chinese). 2017;42(6):711–720. 10.13203/j.whugis20170100.

[29] Lefever DW. Measuring geographic concentration by means of the standard deviational ellipse. The American Journal of Sociology. 1926;32(1):88–94. 10.2307/2765249.

[30] Li XX, Liu DH. Spatial distribution characteristics and evolution trend of China’s marine scientific research institutions. Science Research Management (in Chinese). 2018;39(S1):317–325. 10.19571/j.cnki.1000-2995.2018.ZK.041.

[31] Bai JY. A Study on the Legal Issues of the Arctic Navigation. Beijing: People’s Publishing House; 2016.

[32] Zhao L. Reviewing the multilateral governance paradigm from the perspective of Arctic waterway—A case of the process and practice of “selective compromise” among multiple actors. Journal of International Relations (in Chinese). 2014;(4):63–74. https://doi.org/CNKI:SUN:GGXY.0.2014-04-006.

[33] Li ZW, Gao JT. Analysis about the legal problems of navigation in Arctic. Law Science Magazine. 2010;31(11):62–65. 10.16092/j.cnki.1001-618x.2010.11.034.

